# Early Career South African Occupational Therapists' Perceptions of Person–Environment Fit in Work–Life Areas That Influence Work Stress

**DOI:** 10.1155/2024/3189429

**Published:** 2024-10-23

**Authors:** Patricia de Witt, Morgann Bruce, Marica Botha, Denise Franzsen

**Affiliations:** ^1^Department of Occupational Therapy, School of Therapeutic Sciences, Faculty of Health Sciences, University of Witwatersrand, 7 York Rd., Parktown, Johannesburg, Gauteng, South Africa; ^2^Buckinghamshire Healthcare NHS Trust, Executive Office, Hartwell Wing, Stoke Mandeville Hospital, Mandeville Road, Aylesbury, Buckinghamshire HP21 8AL, UK

## Abstract

**Introduction:** Poor fit between the work environment and work expectations is associated with high levels of workplace-related stress. Work stress in occupational therapy has been attributed to the nature of the profession and various other workplace-related factors. Workplace-related stress leading to burnout has been found to be higher in early career occupational therapists with less than 5 years of experience. This study explored workplace factors that result in poor person–work environment fit in six work–life areas in South African early career occupational therapists.

**Methods:** A descriptive electronic survey design was used to access a sample of South African 261 occupational therapists with 2–5 years of work experience. The Area of Work–Life Survey (AWS) considers six work–life areas: control, workload, community, rewards, values, and fairness, which were used to collect data. Data were analysed to determine low, moderate, and high work environment fit for workplace factors including employment setting, field of practice, years' experience, time with current organization, and hours worked.

**Results:** Of the six work–life areas, only the scores for workload fell below the norm for the respondents. All six work–life areas were impacted by employment settings, with respondents in public health hospitals and clinics indicating significantly low person–environment fit in most areas. Respondents in physical rehabilitation had a significantly low fit for workload, as did years of experience, length of time with organization, and hours worked. Fairness had a low fit for physical rehabilitation and medicolegal fields of practice.

**Conclusion:** Workload impacted workplace-related stress in respondents with scores below the norm on the AWS for this work–life area. Although some work-related factors affected workplace-related stress, the overall scores for the other work–life areas were above the norm, with only respondents in public service settings and in the field of physical rehabilitation reporting low work environment fit.

## 1. Introduction

The World Health Organization (WHO) has described work-related stress as “the response people may have when presented with work demands and pressures that are not matched to their knowledge and abilities and which challenge their ability to cope [1].” The many factors that contribute to workplace-related stress need to be recognized if burnout is to be prevented [2]. Two researchers, Leiter and Maslach [3] in 2003, recognized that the two critical work–life areas that may act as precursors to the development of high workplace-related stress were control over the job and a manageable workload. They also identified a further four work–life areas as potential stressors, namely, the workplace community, workplace rewards, values, and fairness. The workplace community encompasses interpersonal conflict and social support [4], while the power of reinforcement, which shapes behaviour at work, reflects rewards [5]. Values are manifest in beliefs and emotions in relation to job goals, while expectations and equity in the workplace represent fairness [3].

Chronic workplace-related stress has been associated with job–person incongruity, which for healthcare workers results in negative feelings towards their job influencing patient care as well as productivity, efficiency, and effectiveness in the workplace [2, 3]. The stress may also affect engagement in other daily life activities [6]. Occupational therapists in South Africa, like many other healthcare workers, are reportedly exposed to a mismatch between their work environment and their work expectations [7], strongly associated with workplace-related stress [8]. Joshi et al. [9] in 2022 reported that workplace-related stress is exacerbated by an imbalance between job demands and the available resources, both internal and external.

Ongoing workplace-related stress in occupational therapy has been attributed to the nature of the profession which includes exposure to high levels of patient and caregiver distress, uncertain patient outcomes [10], and feelings of being undervalued [11]. These issues are associated with incongruity in the work environment between the perceived expectations and actual experience of social support, workload, rewards, professional identity, and recognition [12, 13]. A lack of effective management, including staffing levels, time and caseload management, availability of resources, and supervision levels, also play a role [14].

Pilkington et al. [15] in 2020 found that professional and workplace-related stress leading to burnout was higher in younger, early career occupational therapists with less than 5 years of experience who had regular employment. This concurs with research which reports occupational therapists with 1–5 years of experience are more likely to leave the profession. Therefore, from a workforce management perspective, it is important to understand the work–life factors that contribute to workplace-related stress of early career professionals who are beginning their work journey, particularly as many occupational therapists in South Africa report high levels of workplace-related stress [12, 16, 17].

## 2. Literature Review

Some stress in the work environment has been reported to be beneficial in improving performance and proficiency; however, when stress becomes overwhelming, it has a negative impact on both an individual's health and work performance [12]. Factors that have been related to occupational stress in healthcare workers include limited work experience, workplace conflict, violence, discrimination, and bullying [18]. Job insecurity and working in a rural or economically deprived place with poor infrastructure and not being sufficiently rewarded or acknowledged for work undertaken also result in workplace stress [9].

Workplace-related stress has been associated with work overload including high levels of overtime and rapid patient turnover, for healthcare professionals. Rising healthcare costs have led to a “do more with less syndrome,” which has led to higher workloads [19] due to the rising burden of disease, especially in low- and middle-income countries, resulting in a shortage of skilled healthcare professionals [20].

There is a global shortage of occupational therapists even in high-income countries [21]. In South Africa, the shortage of occupational therapists in the public healthcare sector has been impacted by poor retention, lack of career paths, and freezing of vacant posts [22]. This has resulted in intensified workloads leading to work-related stress [23]. Clouston in her study in 2014 found occupational therapists reported that more was expected from them at work with limited recovery time and energy for personal and other activities leading to increased stress. Her participants knowingly compromised time for family and other personal daily occupations to achieve obligatory commitments at work, putting others' needs above their own [24]. These pressures were exacerbated by limited control to make changes in the workplace [23].

A lack of job control or the amount of discretion employees have over what they do [25] is associated with an absence of direction, inhibiting work roles and actions within the workplace. Conflict related to expectations in relation to autonomy impacts the sense of control workers have over the job. Occupational therapists in Sweden [24] and the United Kingdom [23] indicated that they felt their control at work and their ability to make changes in their work setting [3, 26] was affected by professional self-doubt and conflict between their perceived role and others' expectations. Globally, occupational therapists report needing to justify their outcomes to other professionals, funding organizations, and patients since their role was frequently misunderstood [12, 27].

A mismatch is evident between occupational therapy outcomes as listed in the Occupational Therapy Practice Framework 4 (OTPF4) [28] which is aligned to the International Classification of Functioning Disability and Health (ICF) [26] and most organizational health outcomes. Occupational therapy outcomes emphasise a biopsychosocial approach including activity and participation, while other health outcomes are measured in relation to body structure and function or as mortality rates, increased in life expectancy, burden of disease, or modified risks [29]. In South Africa, this medical model focus also seems to have been incorporated into the recent shift to support universal healthcare, with rehabilitation as an integrated component of comprehensive healthcare provision being excluded [30]. This proposed healthcare reform and move towards a single health financing model in a National Health Insurance (NHI) has been reported to have added to the work-related stress due to uncertainty and perceived job insecurity for rehabilitation professionals [31].

The lack of resources found in public healthcare settings in many countries, including South Africa, affects work expectations and is linked to workplace-related stress [32]. The South African healthcare system, shaped by its diverse health needs and economic and sociopolitical history, has resulted in a public system that serves more than 80% of the population with limited infrastructure, resources, and staffing [33]. Occupational therapy services face high patient loads and limited access to the resources and equipment needed for therapy and continuous patient care including assistive devices [34].

High workplace-related stress is also commonly associated with a breakdown of the workplace community and is reported to lead to decreased teamwork, undesirable workplace relationships including bullying, professional disrespect, unsupportive and ineffective leadership, and an overall poor workplace environment [35]. Poor communication in the workplace leads to unilateral decision-making, distrust in an organization, and workplace-related stress that may contribute to burnout [35]. A sense of community with colleagues and social support allows buffering of workplace-related stress as people use their social resources to cope with work demands, although high social demands in the workplace may have the opposite effect [3]. Social demands along with unsatisfactory monetary and institutional rewards are additional factors affecting stress at work. These include limited recognition from recipients of the service, others in the workplace, and external stakeholders [3]. Early career occupational therapists have reported inadequate rewards such as lack of recognition from patients and lack of a career path as more important than financial rewards in causing workplace stress [32].

Rewards need to be supported by equity and fairness in the workplace, accommodating the diverse abilities and responsibilities of all workers with the allocation of resources and opportunities, recognition, and rewards [3]. A fair workplace leads to trust, respect, and openness between the organization and service providers. Individuals are reported to be less stressed and more productive if they share responsibility and collaborate to achieve fair and equitable organizational outcomes. Conversely, conflict in the workplace demands time and energy from an individual worker and leads to dissatisfaction and decreased productivity [35].

Work-related stress is also associated with a mismatch between personal expectations and values (a person's ideals and motivations) and workplace organization, structure, and environment [36], resulting in the compromising of personal values [23]. Studies in Australia [37], India [27], and Sweden [24] confirmed these findings with occupational therapists indicating the lack of recognition and the inability to perform their job in accordance with the profession's philosophy [27]. They report a gap between the work the therapists want to do and the work they have or are expected to do [37]. Occupational therapists reported fearing repercussions and feeling compelled to meet organizational demands where contradictory demands or incongruent values resulted in ill health due to workplace-related stress [23].

Personal values and interests also attract individuals to a specific health profession. This results in the profession's mission contributing to an individual's personal goal, allowing for meaningful engagement. However, due to the high competition for medically related education programmes, sometimes individuals end up in medical education programmes that are not necessarily their first choice [38]. This may result in personal and professional values being misaligned, leading to individuals having to sacrifice their needs and wants to satisfy the profession's philosophy [35]. This type of conflict depletes energy, reduces engagement in work, and undermines professional achievements.

This study was aimed at exploring which workplace factors result in poor person–work environment fit in six work–life areas in the early career of South African occupational therapists.

## 3. Materials and Methods

### 3.1. Research Design

As part of a larger study, including burnout [39], a descriptive quantitative, cross-sectional, electronic survey design was used [40] which allowed the researcher to access a national sample of South African occupational therapists with 2–5 years of work experience, irrespective of place, sector, and field of practice, which were the inclusion criteria for this study.

### 3.2. Research Context

The research was completed with occupational therapists working in the South African healthcare, education, corporate, and social services sectors. All therapists complete a university-based training of 4 years in English and are registered with the Health Professions Council of South Africa (HPCSA) [41]. Occupational therapy services in the health sector are divided between an underresourced public sector catering for the majority of the population and a well-resourced private sector catering for less than 20% of the population [34]. Therapists in the public sector are employees for whom the conditions of employment include a 40-h working week. In the private sector, therapists are frequently self-employed and work hours that make their practice financially viable, with no restriction on working hours [42].

### 3.3. Research Population and Sampling

The estimated population of early career occupational therapists was 885, according to the HPCSA. Based on this population using Cochrane's formula with a 5% margin of error, a sample of 269 respondents was required [43]. Nonprobability convenience and snowballing sampling were used [40]. Occupational therapists with 2–5 years of work experience who were members of the Occupational Therapy Association of South Africa or who were on the National Occupational Therapy Forum mailing list were invited to participate in the study. The survey was also made available on South African occupational therapy social media platforms, and respondents were requested to forward the study to other occupational therapists who met the inclusion criteria [40].

### 3.4. Research Process and Data Collection

The researcher designed a demographic questionnaire that provided quantitative data from 12 questions including age, gender, qualification, and work experience. The second data collection tool was the Area of Work–Life Survey (AWS) [44] which rated item statements using a 5-point Likert scale, 1 = s*ubstantial mismatch between the person and their work environment* to 5 = *a strong match between the person and their work environment*. Areas measured included workload (five items), control (four items), reward (four items), community (five items), fairness (six items), and values (four items) [44]. The AWS Cronbach alpha values have been reported to range between 0.70 and 0.85. The correlations of the six areas range between 0.51 and 0.62, indicating that the individual subtests are similarly responsive to work-setting qualities. Criterion-related validity found a strong correlation between the AWS and the Maslach Burnout Inventory: Human Services Survey (MBI-HSS) [45]. The survey developers established normative scores on an international sample of *n* = 22,582 [43]. The AWS has been used in South Africa by other health professionals and is accepted as a suitable measure of work-related stress [46, 47].

The electronic survey on REDCap included the approved information sheet which explained the purpose of the research, and consent was obtained from respondents before they could complete the survey. This research was approved by the Human Research Committee of the University of the Witwatersrand (M190914).

### 3.5. Data Analysis

Descriptive statistics were used to analyse the demographic data and the six work-life areas of AWS using means and percentiles. Data were also compared to the normative data for the AWS using *t* tests and effect sizes. Person and work environment fit was analysed according to the 25th, 50th, and 75th percentile scores as low, moderate, or high. The nonparametric ANOVA Kruskal–Wallis *H* test was used to determine if any statistical significance existed between the percentage of respondents reporting person and work environment fit for the six areas of the AWS and the work-life factors. Only the results where statistical significance was found (set at *p* ≤ 0.05) have been presented.

## 4. Results

A sample of 261 surveys was analysed from the 447 responses returned. Incomplete responses (*n* = 184) were discarded, and two further responses were removed as the respondents did not meet the inclusion criteria.

Most respondents were female (99.23%), and the highest percentage of respondents by age were between 26 and 30 years old (54.40%), and 87.3% of respondents had only completed their first professional qualification. Over a third of respondents had 5 years of experience (39.85%; *n* = 104), and half reported working in the field of paediatrics (52.50%). Half the respondents worked in the private sector (50.96%;*n* = 133) and those who reported "other" as an employment setting worked within the insurance industry and in full or part time academic positions. Most respondents reported working 4–8 h a day (66.67%) and held a general employee status in the organization in which they worked (68.20%) (Table 1).

### 4.1. Workplace Factors

Using the normative data, the authors of the AWS established that a mean score of more than 3 indicated a good work-person fit, while a score below 3 implied there was a mismatch between the person and work environment (Table 2).

Results indicate that for five of the areas of work–life including control, reward, community, fairness, and values, the mean score was above 3, which indicated a balance between areas and the respondents in their workplace. The means were significantly higher than the normative data, indicating a positive work fit in the area of control. Only for the area of *reward* was the mean score not significantly different from the normative score, suggesting a less positive fit for recognition of contribution being acknowledged in the workplace. The overall mean of 2.65 in the *area of workload*, however, indicated a substantial mismatch between the respondents and environment requirements as the mean score was significantly lower than the normative score, indicating that the respondents were experiencing a high workload volume and, thus, higher workplace demands than the normative data sample. The effect sizes between the normative scores and those for the current study were small, indicating little clinical difference between the samples except for values.

**Figure 1:**
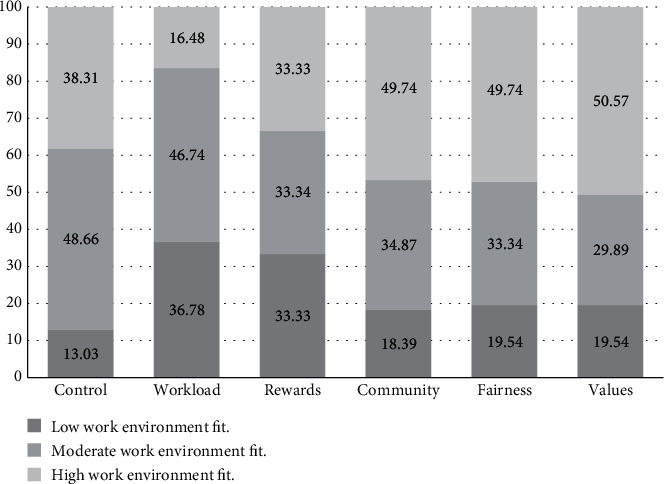
Respondents who experienced low, moderate, and high work environment fit (*n* = 261).

As seen in Figure 1, half of the respondents felt that in the areas of values, community, and fairness, there is a high congruence between themselves and their workplace. Workload and control were the work-life areas where just less than half of the respondents rated a moderate work environment person fit, with a third of respondents indicating a low work environment person fit for workload and rewards.

### 4.2. Workplace Factors Affecting Fit to the Workplace Environment

#### 4.2.1. Employment Setting

Kruskal–Wallis ANOVA indicated there were statistically significant differences for the employment settings and all six areas of the AWS (Table 3). The AWS *workload* differed significantly for different work settings, and respondents working within a public clinical setting (100%), private rehabilitation centres (85%), and schools (75%) experienced the lowest congruence for workload and work environment fit. Respondents working in private hospitals and both a public and private setting reported experiencing a good workload workplace environment fit (71.43%).

Therapists working in a public health clinic (66.67%) were experiencing the least amount of *control* within their workplace environment, indicating a negative person–work environment fit. Respondents working in schools (82.1%) and public rehabilitation centres (80%), as well as over 70% of respondents in private practice, public hospitals, and NGOs, reported high *control* in workplace environment fit.

For the AWS area of *reward*, over 60% of respondents who worked in both private practice and private hospital settings and those working in both private and public settings (71.4%) reported a high workplace fit, while those working in public clinics (100%) experienced the lowest fit.

Three-quarters of respondents in seven employment settings reported a high score for sense of *community* in their workplace environment with private hospitals (92.86%) and two or more private practice settings (89.47%) having the highest percentages. Public clinics experience the least sense of community.

For the *value* area of the AWS, over 70% of respondents in eight employment settings reported congruence between workplace and organization values and their own personal values. The highest percentage was for those working in private hospital settings (85.71%) or two or more private practice settings (85.96%). Respondents in public clinics experienced the lowest work environment fit in terms of values.

#### 4.2.2. Field of Practice

The work environment fit only differed significantly for two areas on the AWS for the field of practice. Respondents in the medicolegal field of practice (83.33%) and mental health (70.37%) experienced the highest *reward* workplace fit, and those in other fields of practice such as the insurance industry reported low rewards. The fields of vocational rehabilitation (100%) and paediatrics (70.27%) were those where the greatest percentage of respondents experienced a high work environment fit for fairness, while 50% or more in physical rehabilitation, medicolegal, and working in two or more fields reported fairness in the workplace environment fit was low (Table 4).

#### 4.2.3. Years of Experience, Number of Hours Worked, and Time at the Current Organization

Only the area of workload on the AWS that differed significantly was for years of experience. Respondents with 5 years of experience (67.31%) reported higher perceived workload and low fit in the workplace environment. The percentage of respondents who reported workload as an issue increased as years of experience increased (Table 5).

The area of workload also differed significantly for the number of hours worked. Respondents working more hours in a day experienced lower fit between their workload within their workplace environment, with the greatest percentage (76.2%) working more than 9 h having a low fit. A statistically significant difference was found for the variable time at the current organization and AWS area of workload. Respondents (73.1%) who had been at their organization for 3–4 years were experiencing the lowest fit for their workplace environment in terms of workload.

## 5. Discussion

The sample represented approximately 29.49% of the estimated population of early career occupational therapists in South Africa, which met the required sample size estimation using the survey table [43]. The demographics of the sample reflect the percentage of early career occupational therapists in South Africa in terms of gender and therapists working in the private and public sectors, as indicated by Ned et al. [22].

In the six areas of work–life on the AWS, the score for the fit to the workplace environment was significantly above the normative values for control, community, values, and fairness. While reward had shown a significant difference from the normative values, the score for workload was significantly lower, indicating incongruency in the workplace environment.

### 5.1. Workload

Workload appears to be the single factor having the greatest impact on work stress in South African occupational therapists, with only 16% of respondents reporting high workplace fit for workload. These findings are supported by similar findings for junior registrars in South Africa [46] and may reflect the estimated global shortage of healthcare workers reported in the WHO Global Strategy on Human Resources [48] which accounts for 69% of the healthcare worker shortage and very limited rehabilitation services [21]. du Plessis, Visagie, and Mji [16] in this study which included occupational therapists in South Africa also found workload was viewed by over 71% of the participants as either above average or overwhelming. Patient load, administration, research, and meetings made up the greatest components of workload, with only 50% of respondents reporting they could achieve deadlines. South African occupational therapists, in a study by Clarke [12], reported supervising students and facilitating training, as well as the obligation to attend events to maintain HPCSA accreditation for continuing professional development [49], which also added to their workload, the high patient caseload being the most problematic. Private rehabilitation centres in South Africa also indicated a high patient-to-therapist ratio and high expectations in terms of documentation by medical insurers [16]. Similar issues were reported internationally for occupational therapists in the United Kingdom, where writing reports after hours was justified so as not to compromise patient care [23]. Teo, Xu, and Ho [50] also reported workload was the single area on the AWS which was significantly associated with burnout in rehabilitation professionals, including occupational therapists, working in Singapore.

In terms of employment settings, ≥ 50% of respondents in eight out of 11 settings indicted low workplace environment fit as a result of workload, with only respondents working in private hospitals, public rehabilitation centres, and employed in both the public and private sectors indicating a higher workplace environment fit. This concurs with the study in Denmark that also reported high workload and overcommitment by occupational therapists working in municipal and council clinics [32].

Low workplace environment fit in relation to workload became more prevalent with increasing years of experience, the length of employment with their current organization, and the more hours a day worked. These findings supported those of Teo, Xu, and Ho [50], who found workplace-related stress was five times more likely to be experienced by younger rehabilitation professionals employed for more than 3 years in their current organization than those with less than 1-year service. Longer duration of employment resulted in greater responsibilities, a higher caseload [50], and an inability to complete required work within expected working hours.

### 5.2. Control

Literature on the AWS suggests that control is the central area in work–life, impacting all other areas. The means and percentiles for control in the current study were higher than the norm for all employment settings except for public hospitals and clinics. A positively perceived capacity under control in private practice, rehabilitation centres, NGOs, and schools may indicate that higher personal autonomy, social support, and rewards in these settings may shield therapists from the stress caused by a high workload [51].

The poor workplace environment fit regarding control in the public health sector may relate to the emphasis on the medical model with little support for rehabilitation, affecting decision-making over intervention, patients' hospital discharge, and continuity of care as well as other workplace responsibilities [27]. This may also reflect the complex South African healthcare system with two unequal health sectors, with private healthcare having more physical resources but less perceived job security due to the funding available and the declining percentage of the population who support this sector [33].

### 5.3. Rewards

Results of the current study indicated similar scores for rewards than the normative data, indicating a reasonable workplace environment fit, with more than two-thirds of respondents reporting a moderate to high congruency of rewards with their workplace. Rewards, which are strongly associated with efficacy at work [27], have been found to be significantly correlated with job satisfaction in occupational therapy and are seen to be a mitigating factor for workplace-related stress and burnout [52]. Working with clients has been reported as the most rewarding part of the job [53]. Scanlan and Still [52] found other rewards such as recognition by colleagues, managers, and patients, and opportunities for professional growth and further skill development [32] were considered more important than remuneration [52].

Respondents in public healthcare clinics and hospitals reported the lowest workplace environment fit with regard to rewards, with a lack of opportunity for developing a career path [31] and lower remuneration [18, 53]. Some concern was expressed by approximately 40% of respondents in physical rehabilitation and other fields of practice in relation to rewards. The acute nature of physical rehabilitation may affect the perception of rewards, as this field of practice is often entrenched within the medical model with limited awareness and recognition from the medical team of the role of rehabilitation and occupational therapy in particular.

### 5.4. Community

Community within the occupational therapy workplace was evaluated by respondents as above the norm on the AWS for nearly half of the sample. These findings are similar to those reported by occupational therapists by Gupta et al. [27], who found that a supportive team and supportive colleagues at work were a protective factor [54]. A lack of support from organizations, inflexible leadership, and a lack of communication within the rehabilitation team were all reported to counteract support and contribute to work-related stress [55].

Respondents working in public clinics reported a low workplace environment fit for the community which may result from the lack of recognition [21] and therefore the lack of integration of rehabilitation into primary healthcare teams at clinics, where the role of the occupational therapists in affecting social determinants of health and lifestyle interventions is rarely appreciated [56].

### 5.5. Fairness

The factors associated with fairness often relate to a lack of recognition of expertise linked to qualifications, further training needs, as well as equitable consideration of all staff opinions [56] and even-handed distribution of work amongst staff. More than half the respondents working in public hospitals and clinics reported a low workplace environment fit for fairness, as did those working in the fields of physical rehabilitation and medicolegal practice. Occupational therapists receive limited recognition and are provided with less resources within the public healthcare system [33]. Low workplace environment fit may be associated with a single national indicator for rehabilitation being the number of wheelchairs issued [57] and prioritizing the volume over effective patient outcomes, as well as the lack of resources needed to offer adequate care [58]. The lack of resources and high workload have resulted in less experienced occupational therapists perceiving they are not able to provide adequate services [21] since it is difficult for them to adapt to and cope with or compensate for this perceived unfairness [58].

The lack of clear professional boundaries between occupational and physiotherapists has been reported as a concern affecting work distribution and blurring of roles in addressing function in rehabilitation [12].

### 5.6. Values

The perceptions of rewards, community, and fairness at work are integrated by the fit of perceived values in the workplace environment [5]. The results for values were therefore in line with those for the areas of rewards, community, and fairness, with more than 50% of respondents reporting an adequate fit between their workplace environment and their values.

The low workplace environment fit was reported by a higher percentage of respondents in the workplace setting of public hospitals and clinics. The low workplace fit reported for these work settings where early career occupational therapists may find developing professional identity is impacted by a lack of inter-professional practice as well as aligning patient outcomes with a strong curative approach used in public healthcare in South Africa [59].

## 6. Limitations of the Study

Some additional open-ended questions in the demographic questionnaire may have given the researcher the opportunity to view the results in more depth considering the South African two financial and resources-inequitable health systems (private and public) that may influence workplace person–environment fit in work–life areas that influence work stress.

By analyzing work stress in various work settings, small samples resulted, and findings should be viewed in this light.

Data for the study were collected at one moment in time; perhaps a longitudinal study with more than a single data collection period may have identified perceptions of workplace factors of a temporal nature.

## 7. Conclusions

This study contributed to the understanding of the environmental factors that contribute to workplace stress which may potentially contribute to burnout in inexperienced early career occupational therapists in South Africa. The workload was identified as the one factor causing stress across all work settings, with a low workplace environment fitting in eight out of 11 work settings. The results also suggest that in the private sector experience and time in employment within an organization and the increase in the hours worked, especially related to administration and practice management, added to work stress. Overall scores for the other work–life areas, namely, control, reward, community fairness, and values, indicted higher workplace environment fit, with a lower impact on stress with the exception of occupational therapists working in physical rehabilitation and medicolegal practice.

## Figures and Tables

**Table 1:** Field of practice, employment setting, years of experience, and hours worked (*n* = 261).

	**n** ** (%)**
Field of practice	
Two or more fields of practice	160 (61.30)
Paediatrics	137 (52.50)
Physical rehabilitation	112 (42.91)
Mental health	71 (27.20)
Vocational rehabilitation	45 (17.24)
Other	31 (11.88)
Medicolegal	25 (9.58)
Employment setting	
Private practice	133 (50.96)
Two or more settings	89 (34.10)
School	52 (19.92)
Public hospital	48 (18.40)
Private hospital	45.(17.24)
Private rehabilitation centre	38.(14.56)
Other	13 (4.98)
NGO	11 (4.21)
Public rehabilitation centre	6.(2.30)
Public clinic	4 (1.53)
Years of work experience	
5	104 (39.85)
4	46 (17.62)
3	68 (26.05)
2	43 (16.48)
Hours per day worked	
9+	84 (32.18)
4–8	174 (66.67)
< 4	3 (1.25)
Time at the current organization	
5 years	48 (18.39)
4 years	26 (9.96)
3 years	53.(20.31)
2 years	69 (26.44)
1 year	65 (24.91)

**Table 2:** Normative data and respondent mean on the Area of Work–Life Survey (*n* = 261).

**Six areas of work–life**	**Mean (SD)**		**95% CI difference**	**p** ** value**	**Effect size (Cohen's ** **d** **)**
	**Normative data (** **n** = 22,582**)**	**Current study (** **n** = 261**)**			
Control	3.31 (0.80)	3.58 (0.84)	0.17–0.36	0.001⁣^∗^	0.33
Workload	2.96 (0.86)	2.65 (0.81)	−0.41 to −0.20	0.001⁣^∗^	0.37
Reward	3.19 (0.89)	3.26 (0.94)	−0.03 to 0.17	0.206	0.07
Community	3.38 (0.84)	3.57 (0.83)	0.08–0.29	0.002⁣^∗^	0.23
Fairness	2.78 (0.80)	3.10 (0.88)	0.22–0.41	0.001⁣^∗^	0.38
Values	3.24 (0.86)	3.60 (0.85)	0.25–0.46	0.001⁣^∗^	0.42

⁣^∗^Significant at < 0.05.

**Table 3:** Low, moderate, and high work environment fit for employment setting and the six work–life areas (*n* = 261).

**Areas of work–life**	**Workload**			**Control**			**Rewards**			**Community**			**Fairness**			**Values**		
**Employment setting**	**LWF**	**MWF**	**HWF**	**LWF**	**MWF**	**HWF**	**LWF**	**MWF**	**HWF**	**LWF**	**MWF**	**HWF**	**LWF**	**MWF**	**HWF**	**LWF**	**MWF**	**HWF**
	**%**																	
Private practice	59.2	11.3	29.5	14.1	9.9	76.1	28.2	11.3	60.6	18.3	2.8	78.9	28.2	7.0	64.8	12.7	7.0	80.3
Private hospital	28.6	0.0	71.4	7.1	14.3	78.6	21.4	14.3	64.3	7.1	0.0	92.9	21.4	0.0	78.6	7.1	7.1	85.7
Private rehabilitation centre	85.0	0.0	15.0	20.0	20.0	60.0	35.0	10.0	55.0	10.0	15.0	75.0	50.0	10.0	40.0	15.0	25.0	60.0
Public clinic	100	0.0	0.0	66.7	0.0	33.3	100	0.0	0.0	66.7	0.0	33.3	66.7	0.0	33.3	100.0	0.0	0.0
Public hospital	51.2	9.8	39.0	39.0	7.3	53.7	53.7	14.6	31.7	39.0	4.9	56.1	75.6	7.3	17.1	51.2	7.3	41.5
Public rehabilitation centre	40.0	0.0	60.0	0.0	20.0	80.0	40.0	0.0	60.0	20.0	0.0	80.0	40.0	20.0	40.0	20.0	0.0	80.0
NGO	50.0	0.0	50.0	25.0	0.0	75.0	0.0	50.0	50.0	25.0	25.0	50.0	0.0	0.0	100	25.0	0.0	75.0
School	75.0	3.6	21.4	17.9	0	82.1	28.6	17.9	53.6	17.9	14.3	67.9	46.4	3.6	50.0	10.7	10.7	78.6
Other	50.0	0.0	50.0	20.0	20.0	60.0	20.0	10.0	70.0	10.0	0.0	90.0	40.0	0.0	60.0	20.0	10.0	70.0
Two or more private practice settings	63.2	12.2	24.6	10.5	5.3	84.6	28.1	7.0	64.9	7.0	3.5	89.5	33.3	3.5	63.2	8.8	5.3	86.0
Both public and private	28.6	0.0	71.4	28.6	0.0	71.4	28.6	0	71.4	14.3	0.0	85.7	28.6	0.0	71.4	28.6	0.0	71.5
*p* value	0.009			0.021			0.003			0.003			0.003			0.001		

Abbreviations: HWF = high work environment fit (percentage), LWF = low work environment fit (percentage), MWF = moderate work environment fit (percentage).

**Table 4:** Low, moderate, and high work environment fit for the field of practice and work–life areas (*n* = 261).

**Areas of work–life**	**Reward**			**Fairness**		
**Field of practice**	**Low work environment fit (%)**	**Moderate work environment fit (%)**	**High work environment fit (%)**	**Low work environment fit (%)**	**Moderate work environment fit (%)**	**High work environment fit (%)**
Paediatrics	28.2	6.8	66.2	22.9	6.8	70.3
Mental health	18.5	11.1	70.4	40.7	11.1	48.2
Physical rehabilitation	40.9	13.6	45.5	50.0	9.1	40.9
Medicolegal	0.0	16.7	83.3	50.0	0.0	50.0
Vocational rehabilitation	33.3	0.0	66.7	0.0	0.0	100.0
Other	42.9	28.6	28.6	42.9	14.3	42.9
Two or more fields	40.0	13.0	66.2	52.0	2.0	46.0
*p* value	0.030			0.006		

**Table 5:** Low, moderate, and high work environment fit for years of experience, number of hours worked, time at the current organization, and work–life areas (*n* = 261).

**Area of work–life**	**Workload**		
	**Low work environment fit (%)**	**Moderate work environment fit (%)**	**High work environment fit (%)**
Years of experience			
2 years	41.9	11.6	46.5
3 years	55.9	5.9	38.2
4 years	65.2	8.7	26.1
5 years	67.3	1.9	26.0
*p* value	0.027		
Number of hours worked			
Less than 4 h	25.0	0.0	75.0
4–8 h	53.3	10.7	39.1
9 or more hours	76.2	2.4	21.4
*p* value	0.002		
Time at the current organization			
Under 6 months	52.9	11.8	35.3
6 months–1 year	48.4	12.9	28.7
1–2 years	58.0	7.3	34.8
2–3 years	66.0	3.8	30.2
3–4 years	73.1	7.7	19.2
4–5 years	60.4	6.3	33.3
*p* value	0.008		

## Data Availability

Data is available from the second author and is stored in a REDCap repository at the University of the Witwatersrand.
